# Exercise attitudes and practices among adults listed for kidney transplantation: a survey of a diverse patient cohort

**DOI:** 10.3389/fspor.2025.1559322

**Published:** 2025-07-14

**Authors:** Sadia Tasleem, Kamlesh Patel, James Hodson, Farah Mazhar, Laura Bedford, Felicity R. Williams, Simon Jones, Matthew J. Armstrong, Adnan Sharif, Dilan Dabare

**Affiliations:** ^1^ Department of Renal Transplant Surgery, University Hospitals Birmingham NHS Foundation Trust, Birmingham, United Kingdom; ^2^ Institute of Inflammation and Ageing, College of Medical and Dental Sciences, University of Birmingham, Birmingham, United Kingdom; ^3^ Department of Research and Innovation, Queen Elizabeth Hospital Birmingham, Birmingham, United Kingdom; ^4^ Institute of Immunology and Immunotherapy, College of Medical and Dental Sciences, University of Birmingham, Birmingham, United Kingdom; ^5^ Department of Inflammation and Ageing, NIHR Birmingham Biomedical Research Centre, Birmingham, United Kingdom

**Keywords:** exercise, kidney transplantation, prehabilitation, attitudes, waiting list

## Abstract

**Background:**

Given the increasing age and frailty of kidney transplant candidates, there is an emerging drive to optimise patients before transplantation. Lack of exercise has been linked with poor outcomes at all stages of the transplant pathway. The aim of this study was to evaluate the attitudes and perception to exercise in such patients and assess how these practises vary by demographics.

**Methods:**

A single-centre, prospective, survey-based study was conducted on consecutive adult patients being assessed for activation on the deceased-donor kidney transplant waiting list.

**Results:**

A total of 103 patients (65% male; 56% White ethnicity; mean age: 47.8 years) completed the survey. Of these, 42% were on haemodialysis and 24% on peritoneal dialysis. Most patients agreed/strongly agreed that exercise was important (86%) and that they would be willing to do so to optimise their health (97%). Despite this, only 56% of patients reported exercising on a regular basis. Most patients stated that they would be willing to wear exercise monitoring devices (81%). Younger (Spearman's rho: 0.20, *p* = 0.047) and Black/Asian ethnicity (*p* = 0.038) patients reported performing significantly less exercise activity than their older and White counterparts.

**Conclusion:**

Whilst kidney transplant candidates have generally positive attitudes toward exercise, only around half of those surveyed reported exercising regularly. The findings of this study, including differences across age and ethnicity, would be useful to consider when designing patient-centred prehabilitation interventions to encourage exercise in this cohort.

## Introduction

Chronic kidney disease (CKD) is a growing public health emergency, due to an ageing and more co-morbid population (1). These factors are contributing to an increasing burden of physical frailty in patients awaiting kidney transplantation, which remains the optimal intervention for end-stage kidney disease (ESKD) (2). Recent UK data has demonstrated that 35%–40% of kidney transplant candidates are either frail or “vulnerable” to frailty (3). Frailty is an age-related, sustained loss in physiological reserve, which is further compounded in kidney transplant candidates by underlying disease progression, associated comorbidities and adverse effects of dialysis (4). At all ages, ESKD patients are more susceptible to developing frailty when compared to the general population (5, 6). The presence of frailty is now well-recognised as impacting on all aspects of kidney transplantation, resulting in a lower likelihood of receiving a kidney transplant, either due to waiting list mortality or being removed from the waiting list, as well as poorer post-transplant patient and graft survival (5, 7).

Studies have shown that frailty and overall health status can be improved through exercise and increased physical activity (8, 9). There is a growing realisation in the transplant community that this form of intervention should be delivered through personalised prehabilitation programs, and this remains an area of active interest (10, 11). The relatively long waiting-list period (averaging two years in the UK) for a suitable kidney graft provides a window of opportunity to deliver prehabilitation (12). An international working group from the European Society for Organ Transplantation (ESOT) recently published a consensus document stating the increasing importance of research into various aspects of prehabilitation programs (13).

Attitudes to, and perceptions of exercise in kidney transplant candidates are poorly understood, particularly in the context of varying demographics such as age and ethnicity. Available evidence is based on the wider population of CKD patients, which is a heterogenous group, with not all being eligible for transplantation (14, 15). Kidney transplant candidates are a select group and an understanding of their unique views and circumstances is paramount in designing future clinical trials, ensuring sustained engagement and achieving positive outcomes. Recruitment, retention, and protocol adherence in exercise trials tend to be poor, suggesting that trial design may not resonate with patients (16). In addition, whilst the use of advanced monitoring and exercise techniques, such as wearable accelerometers and resistance exercises, respectively, have shown some benefit, patient's attitudes to these remain poorly explored.

The aim of this study was to collect data to inform the exercise intervention component of a future prehabilitation trial in patients on the waiting list for a deceased donor kidney transplant. The primary objective was to assess patients' attitudes to three components related to exercise at the time of entry to the kidney transplant waiting list, namely willingness to exercise, current activity levels, and barriers to exercise. The secondary objective was to identify associations between clinical features (e.g., age, ethnicity, gender, and type of dialysis) and both levels of physical activity and willingness to exercise.

## Methods

### Study overview

A single-centre, prospective, survey-based study was performed on adult patients listed for deceased donor kidney transplantation at a high-volume renal transplant centre. Patients were approached at the time of their assessment for activation on the deceased donor transplant waiting list and provided with a patient information sheet and a survey. The patient information sheet stated that participation was optional, and neither the choice to participate nor the answers given would have any influence on treatment; patients were deemed to give consent by completing the survey. The study was registered as an audit and quality improvement project at our institution (CARMS18907).

### Eligibility

All adult patients (age 18 or above) listed for deceased donor kidney transplantation between February and September 2023 were eligible for inclusion in the study. Non-English-speaking patients were assisted to complete the survey by their next of kin, interpreter or the transplant co-ordinator in clinic.

### Data collection

The survey was based on one used in a previous study of patients listed for liver transplantation, and was adapted for use in patients with ESKD (14). It comprised five-point Likert scale questions, to assess patients' attitudes towards exercise; the type and duration of exercise performed; and barriers to performing exercise (see Table 1). The response options for the Likert scale questions relating to agreement were assigned values between 1 = strongly disagree and 5 = strongly agree for analysis, such that higher scores were indicative of greater agreement with the statement.

**Table 1:** Survey questions.

Question		Response options
(A)	It is important for patients with renal failure to exercise whilst awaiting a kidney transplant	Likert^a^
(B)	It is reasonable to expect someone like me to exercise regularly	Likert^a^
(C)	I would be willing to exercise whilst awaiting kidney transplant to optimize my health	Likert^a^
(D)	I would be willing to wear an exercise monitoring device (e.g., Fitbit, Jawbone) to optimise my health whilst awaiting kidney transplant	Likert^a^
(E)	My doctor has encouraged me to exercise more	Likert^a^
(F)	I exercise on a regular basis	Likert^a^
(G)	The number of days per week that I typically exercise are	<1, 1–2, 3, 4, ≥5
(H)	The duration of my exercise sessions are usually (in minutes)	≤10, 20, 30, 40, ≥60
(I)	I regularly perform these activities:	
	Walking	Likert^a^
	Jogging	Likert^a^
	Swimming/water aerobics	Likert^a^
	Stationary bicycling	Likert^a^
	Light resistance training (exercise bands)	Likert^a^
	Weight machines	Likert^a^
	Other (please describe)	Free-text
(J)	Factors limiting my ability to exercise include:	
	Access to exercise equipment/facilities	Likert^a^
	Fatigue	Likert^a^
	Physician recommendation against exercise	Likert^a^
	Medications	Likert^a^
	Time spent on haemodialysis (if appropriate)	Likert^a^, N/A
	Fluid restriction (if appropriate)	Likert^a^, N/A
	Other (please describe)	Free-text

^a^ A five-point Likert scale of: “strongly disagree”; “disagree”; “neither agree nor disagree”; “agree”; and “strongly agree”. N/A, not applicable.

Patient demographic data [age, body mass index (BMI), gender and ethnicity], comorbidities (hypertension, ischemic heart disease, diabetes mellitus, and smoking status), and the current type of dialysis were obtained from individual patient case notes.

### Statistical methods

The Likert scale questions were treated as ordinal for analysis and compared across nominal variables using Mann–Whitney U or Kruskal–Wallis tests for factors with two, or more than two categories, respectively. Associations with ordinal or continuous variables were assessed using Spearman's rank correlation coefficients (rho) and the associated *p*-values. All analyses were performed using IBM SPSS 24 (IBM Corp. Armonk, NY), with *p* < 0.05 deemed to be indicative of statistical significance throughout. Cases with missing data, either due to patients not giving a response or where a question was not applicable, were excluded from the analysis of the affected question. Continuous variables were summarised as “mean ± standard deviation” where approximately normally distributed, or as “median (interquartile range)” otherwise.

## Results

### Patient population

A total of 105 surveys were issued and returned during the study period. Of these, the surveys from two participants were discarded due to lack of completion of the survey, resulting in a total sample size of 103 (98% response rate). The 103 patients who completed the survey had a mean age at listing of 47.8 years, with 65% being male and 56% of White ethnicity. These demographics are in keeping with gender and ethnic variation seen in our regional CKD population (17). The majority had hypertension (82%), with 42% being on haemodialysis and 24% on peritoneal dialysis (PD). The remaining 34% of patients were pre-dialysis, with an estimated glomerular filtration rate (eGFR) < 15 ml/min/1.73 m^2^, who were pre-emptively listed for a deceased-donor transplantation. Further details of the cohort are reported in Table 2*.*

**Table 2:** Participant demographics.

	*N*	Statistic
Age at listing (years)	103	47.8 ± 15.2
Gender (% male)	103	67 (65%)
Body mass index (kg/m^2^)	103	27.4 ± 5.4
Ethnicity	103	
White		58 (56%)
Asian		28 (27%)
Black		13 (13%)
Mixed/other		4 (4%)
Smoking status	103	
Never-smoker		76 (74%)
Ex-smoker		15 (15%)
Current smoker		12 (12%)
Hypertension	103	84 (82%)
Ischemic heart disease	103	11 (11%)
Diabetes mellitus	103	17 (17%)
Clinical frailty score	83	
(1) Very fit		7 (8%)
(2) Well		31 (37%)
(3) Managing well		24 (29%)
(4) Vulnerable		16 (19%)
(5) Mildly frail		3 (4%)
(6) Moderately frail		2 (2%)
Aetiology of end-stage kidney disease	90	
Renovascular/systemic and metabolic disorders		33 (37%)
Glomerular disease		30 (33%)
Congenital/genetic renal disease		14 (16%)
Tubulointerstitial diseases		13 (14%)
Type of dialysis	103	
Not on dialysis		35 (34%)
Haemodialysis		43 (42%)
Peritoneal dialysis		25 (24%)
Dialysis vintage (months)^a^	67^a^	24 (18–48)

Data are reported as “mean ± standard deviation”, “median (interquartile range)”, or “*N* (%)”, as applicable.

^a^ In patients on dialysis, for whom data were available.

### Exercise attitudes and behaviour

**Figure 1:**
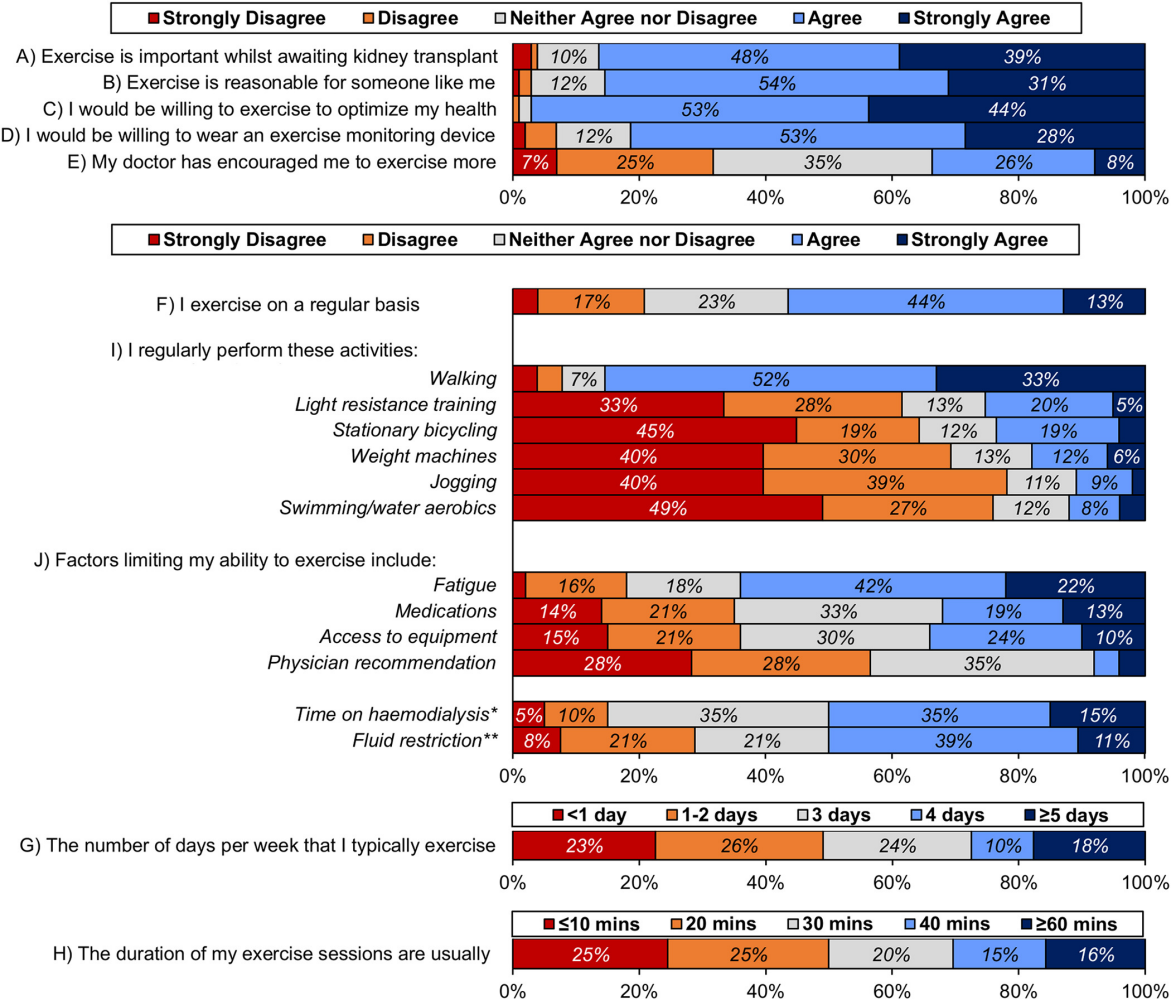
Responses to Likert-scale questions. The wording of some questions is shortened for brevity; the original text of each question is reported in Table 1. Percentages are calculated based on the number of patients that gave a response to the stated question, as reported in Table 3. For questions I) and J), the activities/factors are sorted in descending order, based on the mean response. Unlabelled bars have a frequency of <5%. *For patients on haemodialysis. **Excludes patients answering “not applicable”, i.e., those without fluid restrictions.

Survey responses are reported in Table 3 and visualised in Figure 1. Most patients agreed/strongly agreed that exercise was important whilst awaiting a kidney transplant (86%); that it would be reasonable to expect them to exercise regularly (85%); and that they would be willing to do so to optimise their health (97%). However, despite this, only 56% of patients agreed/strongly agreed that they currently exercised on a regular basis, with the average amount of exercise being approximately two sessions of 20 min per week.

**Table 3:** Responses to survey questions.

Question	*N*	Strongly disagree	Disagree	Neither agree nor disagree	Agree	Strongly agree
(A) Exercise is important whilst awaiting kidney transplant	103	3 (3%)	1 (1%)	10 (10%)	49 (48%)	40 (39%)
(B) Exercise is reasonable for someone like me	103	1 (1%)	2 (2%)	12 (12%)	56 (54%)	32 (31%)
(C) I would be willing to exercise to optimize my health	103	0 (0%)	1 (1%)	2 (2%)	55 (53%)	45 (44%)
(D) I would be willing to wear an exercise monitoring device	102	2 (2%)	5 (5%)	12 (12%)	54 (53%)	29 (28%)
(E) My doctor has encouraged me to exercise more	101	7 (7%)	25 (25%)	35 (35%)	26 (26%)	8 (8%)
(F) I exercise on a regular basis	101	4 (4%)	17 (17%)	23 (23%)	44 (44%)	13 (13%)
(I) I regularly perform these activities:						
Walking	103	4 (4%)	4 (4%)	7 (7%)	54 (52%)	34 (33%)
Jogging	101	40 (40%)	39 (39%)	11 (11%)	9 (9%)	2 (2%)
Swimming/water aerobics	100	49 (49%)	27 (27%)	12 (12%)	8 (8%)	4 (4%)
Stationary bicycling	98	44 (45%)	19 (19%)	12 (12%)	19 (19%)	4 (4%)
Light resistance training	99	33 (33%)	28 (28%)	13 (13%)	20 (20%)	5 (5%)
Weight machines	101	40 (40%)	30 (30%)	13 (13%)	12 (12%)	6 (6%)
(J) Factors limiting my ability to exercise include:						
Access to equipment/facilities	100	15 (15%)	21 (21%)	30 (30%)	24 (24%)	10 (10%)
Fatigue	100	2 (2%)	16 (16%)	18 (18%)	42 (42%)	22 (22%)
Physician recommendation	99	28 (28%)	28 (28%)	35 (35%)	4 (4%)	4 (4%)
Medications	100	14 (14%)	21 (21%)	33 (33%)	19 (19%)	13 (13%)
Time spent on haemodialysis (if on haemodialysis)^a^	40	2 (5%)	4 (10%)	14 (35%)	14 (35%)	6 (15%)
Fluid restriction^b^	66	5 (8%)	14 (21%)	14 (21%)	26 (39%)	7 (11%)
		**<1 day**	**1–2 days**	**3 days**	**4 days**	**≥5 days**
G) The number of days per week that I typically exercise are	102	23 (23%)	27 (26%)	24 (24%)	10 (10%)	18 (18%)
		**≤10 min**	**20 min**	**30 min**	**40 min**	**≥60 min**
H) The duration of my exercise sessions are usually	102	25 (25%)	26 (25%)	20 (20%)	15 (15%)	16 (16%)

The wording of some questions is shortened for brevity; the original text of each question is reported in Table 1. Percentages are calculated based on the number of patients that gave a response to the stated question.

^a^ For patients on haemodialysis.

^b^ Excludes patients answering “not applicable”, i.e., those without fluid restrictions.

By far the most common exercise activity was walking, with 85% of patients agreeing/strongly agreeing that this was performed on a regular basis, this was followed by light resistance training (25%) and stationary bicycling (23%). Free-text responses listing activities not already included in the survey were given by seven patients; these included yoga/Pilates (*N* = 3) and sports (badminton/cricket; *N* = 2). Fewer participants engaged in exercises of increasing intensity.

Fatigue was the most common factor limiting patients' ability to exercise (agree/strongly agree: 64%); time spent on haemodialysis (50%) and fluid restriction (50%) were also major barriers to exercise for the subgroups of patients for whom these were applicable. Only 34% of patients reported that they had been encouraged to do more exercise by their doctor. The majority of patients stated that they would be willing to wear exercise monitoring devices (agree/strongly agree: 81%).

### Associations between patient factors and exercise attitudes and behaviour

**Figure 2:**
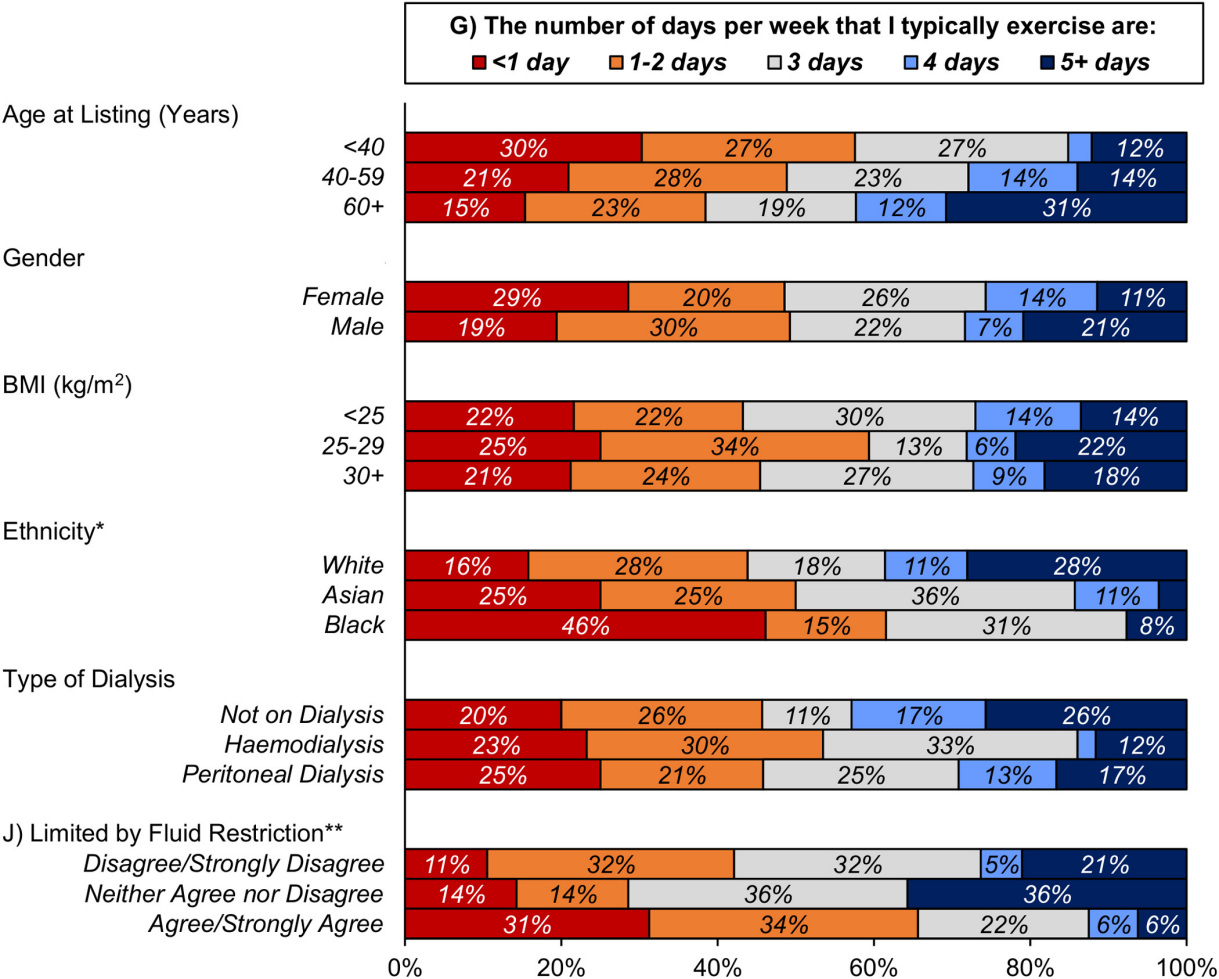
Associations between patient characteristics and exercise frequency. The wording of some questions is shortened for brevity; the original text of each question is reported in Table 1. Percentages are calculated based on the number of patients that gave a response to the stated question, as reported in Table 3. Unlabelled bars have a frequency of <5%. *The mixed/other group was excluded from analysis, due to the small sample size. **Excludes patients answering “not applicable”, i.e., those without fluid restrictions. BMI, body mass index.

None of the patient characteristics considered were found to be significantly associated with views on the importance or reasonableness of exercise, or with willingness to exercise (Table 4A). However, significant associations with the amount of exercise reported by patients were observed (Table 4B; Figure 2). Specifically, older patients tended to perform greater amounts of exercise, with the proportion of patients reporting that they exercise regularly increasing from 44% in those aged <40 years to 76% in those aged 60+ years (rho: 0.24, *p* = 0.016) and a corresponding increase in those reporting exercising on more than three days per week (15% vs. 42%, rho: 0.20, *p* = 0.047). A significant difference across subgroups of ethnicity was also observed, with 39% of White patients exercising on more than three days per week, compared to 14% of Asian and 8% of Black patients (*p* = 0.038). Neither gender, BMI, nor type of dialysis were found to be significantly associated with the views on exercise, or the amount of exercise performed.

**Table 4A:** Associations between patient characteristics and views on exercise.

	(A) Exercise is important		(B) Exercise is reasonable		(C) Willing to exercise	
	% Agree	*p*-value	% Agree	*p*-value	% Agree	*p*-value
Age at listing (years)	*Rho: 0.15*	0.120	*Rho: 0.17*	0.085	*Rho: 0.02*	0.857
<40	29 (88%)		26 (79%)		33 (100%)	
40–59	38 (86%)		37 (84%)		41 (93%)	
60+	22 (85%)		25 (96%)		26 (100%)	
Gender	–	0.196	–	0.496	–	0.504
Female	30 (83%)		31 (86%)		35 (97%)	
Male	59 (88%)		57 (85%)		65 (97%)	
BMI (kg/m^2^)	*Rho: −0.16*	0.115	*Rho: −0.17*	0.091	*Rho: −0.11*	0.254
<25	37 (97%)		34 (89%)		37 (97%)	
25–29	23 (72%)		26 (81%)		31 (97%)	
30+	29 (88%)		28 (85%)		32 (97%)	
Ethnicity^a^	–	0.280	–	0.823	–	0.321
White	52 (90%)		51 (88%)		56 (97%)	
Asian	25 (89%)		23 (82%)		27 (96%)	
Black	10 (77%)		11 (85%)		13 (100%)	
Type of dialysis	–	0.515	–	0.277	–	0.427
Not on dialysis	29 (83%)		31 (89%)		34 (97%)	
Haemodialysis	36 (84%)		36 (84%)		41 (95%)	
Peritoneal dialysis	24 (96%)		21 (84%)		25 (100%)	
(J) Limited by fluid restriction^b^	*Rho: −0.07*	0.554	*Rho: −0.20*	0.107	*Rho: 0.14*	0.278
Disagree/strongly disagree	16 (84%)		18 (95%)		18 (95%)	
Neither agree nor disagree	13 (93%)		14 (100%)		14 (100%)	
Agree/strongly agree	29 (88%)		25 (76%)		32 (97%)	

For nominal variables, the Likert scale questions were compared across categories using Mann–Whitney U test (for gender) or Kruskal–Wallis test (for ethnicity and type of dialysis) – to visualise the trends, the *N* (%) of patients answering agree/strongly agree to the questions are then reported for each subgroup. For ordinal and continuous variables (age at listing, BMI, and fluid restriction), associations with the Likert scale questions were assessed using Spearman's correlation coefficients (rho) – to visualise the trends, the variables were then divided into three subgroups, with the N (%) of patients answering agree/strongly agree to the questions reported for each subgroup. The full text of questions A, B, C and J is reported in Table 1. Bold *p*-values are significant at *p* < 0.05.

^a^ The mixed/other group was excluded from analysis, due to the small sample size.

^b^ Excludes patients answering “not applicable”, i.e., those without fluid restriction. BMI, body mass index.

**Table 4B:** Associations between patient characteristics and amount of exercise.

	(F) Exercise regularly		(G) Exercise days per week		(H) Exercise session duration	
	% Agree	*p*-value	% >3 days^b^	*p*-value	% >30 mins^b^	*p*-value
Age at listing (years)	*Rho: 0.24*	**0**.**016**	*Rho: 0.20*	**0**.**047**	*Rho: 0.11*	0.291
<40	14 (44%)		5 (15%)		10 (30%)	
40–59	24 (55%)		12 (28%)		12 (28%)	
60+	19 (76%)		11 (42%)		9 (35%)	
Gender	–	0.331	–	0.518	–	0.154
Female	19 (54%)		9 (26%)		8 (22%)	
Male	38 (58%)		19 (28%)		23 (35%)	
BMI (kg/m^2^)	*Rho: −0.18*	0.065	*Rho: 0.01*	0.948	*Rho: −0.15*	0.134
<25	23 (61%)		10 (27%)		14 (37%)	
25–29	19 (61%)		9 (28%)		9 (28%)	
30+	15 (47%)		9 (27%)		8 (25%)	
Ethnicity^a^	–	0.685	–	**0**.**038**	–	0.534
White	36 (62%)		22 (39%)		21 (37%)	
Asian	14 (52%)		4 (14%)		8 (29%)	
Black	5 (42%)		1 (8%)		2 (15%)	
Type of dialysis	–	0.487	–	0.337	–	0.414
Not on dialysis	23 (66%)		15 (43%)		13 (37%)	
Haemodialysis	20 (49%)		6 (14%)		8 (19%)	
Peritoneal dialysis	14 (56%)		7 (29%)		10 (40%)	
(J) Limited by fluid restriction^c^	*Rho: −0.13*	0.286	*Rho: −0.33*	**0**.**008**	*Rho: −0.16*	0.214
Disagree/strongly disagree	11 (58%)		5 (26%)		7 (37%)	
Neither agree nor disagree	10 (71%)		5 (36%)		4 (29%)	
Agree/atrongly agree	14 (44%)		4 (13%)		5 (16%)	

For nominal variables, the Likert scale questions were compared across categories using Mann–Whitney U test (for gender) or Kruskal–Wallis test (for ethnicity and type of dialysis) – to visualise the trends, the *N* (%) of patients answering one of the top two Likert categories to the questions are then reported for each subgroup. For ordinal and continuous variables (age at listing, BMI, and fluid restriction), associations with the Likert scale questions were assessed using Spearman's correlation coefficients (rho) – to visualise the trends, the variables were then divided into three subgroups, with the *N* (%) patients answering one of the top two Likert categories to the questions reported for each subgroup. The full text of questions F, G and H is reported in Table 1. Bold *p*-values are significant at *p* < 0.05. BMI, body mass index.

^a^ The mixed/other group was excluded from analysis, due to the small sample size.

^b^ Percentages represent the proportion of patients answering 4/≥5 days, or 40/≥60 min, respectively.

^c^ Excludes patients answering “not applicable”, i.e., those without fluid restrictions.

## Discussion

This survey demonstrates that, whilst ESKD patients who are listed for kidney transplantation have a positive attitude towards exercise, only around half report regularly performing any form of exercise, with fatigue being a major barrier. To our knowledge, this is the first study of attitudes to exercise in kidney transplant candidates and, as intended by the study, provides granular views based on varying demographics, type of dialysis and exercise patterns. Attitudes to exercise varied between different age groups and ethnicities, suggesting the need for personalised interventions. Most patients were willing to consider a wearable device to monitor sedentary behaviour. Only a minority of patients performed any form of resistance exercise.

This study was inspired by Chasca et al., who evaluated patient perspectives regarding exercise in liver transplant candidates (14). They reported similar findings, with around 90% of patients stating that exercise was important and that that they would be willing to exercise, but only around half reporting regularly performing exercise, with walking being the most common. Delgado et al. performed a similar survey on 100 ESKD patients undergoing haemodialysis (18). They also reported findings consistent with our audit, with 54% of patients stating that they did not perform even 30 min of light activity a day. In addition, 8% of patients of their cohort were concerned about the risk of exercise and 24% acknowledged a fear of getting hurt. Whilst we did not specifically ask about concerns relating to exercise, 15% of patients stated that they did not agree that exercise would be reasonable for them, some of which may have reflected similar concerns. Finally, in addition to fatigue, Delgado et al. also noted that “lack of motivation” was a key barrier to exercise – a concept that we did not explore in our study.

Only 34% of our cohort agreed/strongly agreed that their doctor had encouraged them to do more exercise. An international survey of nephrologists by Taryana et al. indicated that most nephrologists viewed exercise counselling as within the scope of their practise and identified exercise prescriptions and programs as an important area of research to further aid counselling (19). However, studies have often found exercise counselling to be poor because of competing interests and fears of harm to the patient (20). MacRae et al. assessed healthcare provider counselling in 108 ESKD patients receiving PD, and found considerable variability in the exercise-related advice provided (15). For example, 76% were told not to lift weights and 44% were told not to swim due to the presence of a PD catheter. This may reflect the lack of clear guidance on exercise in PD patients and requires further evaluation given the discrepancy in patient and clinician views (21).

Less than a quarter of patients in our cohort agreed/strongly agreed to performing resistance or weight training. This is in keeping with the low rate of recruitment seen in trials that have attempted to study resistance training in the CKD population (22). In CKD, skeletal muscle wasting is associated with increased morbidity and mortality, which worsens in ESKD patients who start dialysis. Evidence suggests that resistance training it is well tolerated by CKD patients and confers important clinical benefits (23, 24). However, perceptions of resistance training have only been studied in patients with CKD stage 3 and 4, and not ESKD or kidney transplant candidates (25). Therefore, further work is required to address how to encourage resistance training and how best to deliver programs (e.g., home- vs. hospital-based) whilst waiting for a kidney transplant (26).

We demonstrated a significant difference in physical activity levels between ethnic groups in our cohort, with Black and Asian individuals performing less activity compared to their White counterparts. This was despite there being no significant difference between ethnic groups in the perceptions of, and willingness to exercise. Ethnic minority groups have a greater burden of risk factors for CKD and are 3–5 times more likely to progress to dialysis than White patients. In the UK, over a third of people waiting for a kidney transplant are from ethnic minority communities. Therefore, it is important to reflect on how physical activity interventions can be tailored to these patients (24). Mayes et al. performed a qualitative study exploring the cultural and ethnic influences on physical activity in CKD patients, and concluded that attitudes and beliefs towards exercise vary across ethnic groups (27). For example, in South Asian patients, inter-generational relationships and relatives play an important role in diet and exercise, which needs to be considered when designing exercise interventions.

We did not note any clear differences in either views on exercise or physical activity levels between the subgroups of pre-dialysis, haemodialysis and PD patients. This may reflect heterogeneity in symptom burden that impedes exercise, even amongst end-stage pre-dialysis patients, as they approach dialysis (9). Consequently, dialysis does not appear to have directly impacted physical activity in this cohort. However, half of the patients on haemodialysis reported the time spent on dialysis to be a barrier to exercise, with half of those with fluid restrictions reporting this to be a barrier. As such, these additional challenges may represent indirect impacts on the ability of patients to exercise, which should be considered when designing exercise interventions. For example, programmes of shorter but more frequent sessions may be more convenient for patients who have limited time to dedicate to exercise, whilst lower-intensity exercises could be more appropriate for patients on fluid restrictions.

This study had several strengths, with the main one being that it is unique in the current literature, as it focuses on kidney transplant candidates. Given the wide spectrum of CKD, further studies such as these are required to gain specific understanding of attitudes of CKD patients listed for transplantation, as these may differ from the general CKD population. In addition, unlike most exercise studies that focus predominately on haemodialysis patients alone, this cohort includes pre-dialysis and PD patients making the results more generalisable. The cohort was also diverse, with a large proportion being of non-White ethnicity (44%), an under-represented group in exercise studies; the fact that patients with language barriers were not excluded, and instead supported in completing the survey likely contributed to this diversity. However, our study also has limitations, which need to be considered when interpreting the results. Firstly, the self-reported nature of the study meant that the reliability of the results is dependent on patients answering the questions truthfully and accurately. However, it is possible that social desirability bias may have resulted in some patients giving overly positive responses, particularly in the knowledge that their answers would potentially be seen by their clinician. As such, future studies would ideally use wearable exercise monitoring devices, to validate patients' responses. Secondly, the study had only modest statistical power, resulting from the relatively small sample size. A *post-hoc* power calculation indicated that the sample size of *N* = 103 yielded a minimal detectable correlation coefficient of 0.3 at 80% power. As such, whilst the study was sufficiently powered to detect moderate-to-large effects, small effects may have been missed, leading to false-negative errors.

Thirdly, the survey did not formally define “exercise”, which may have resulted in inconsistency of responses if the interpretation of this term varied between patients. This can be seen by the discrepancy between the proportion of patients stating that they regularly exercised, and that reported walking regularly (agree/strongly agree = 56% vs. 85%), implying that some patients classified walking as exercise and others did not. This may also explain why older patients in our cohort appear to engage in greater amounts of exercise, contrary to other reported studies (18). This confusion between what is considered “physical activity” or “exercise” has been documented in many exercise-related studies (28). As such, this provides further justification for the use of wearable exercise monitoring devices in future studies, to allow for a more consistent measurement of the quantity and intensity of physical activity. Finally, our cohort was relatively young (mean age: 47.8 years), compared to the current European demographic (mean age: 50–52 years) (29, 30). This was particularly pertinent, given that some of the responses (e.g., exercise frequency) were found to differ significantly with age. Consequently, the results may not be generalisable to cohorts with a considerably different age distribution.

## Conclusion

Whilst the vast majority of kidney transplant candidates have positive attitudes towards exercise, a smaller proportion report regularly performing exercise. With rising levels of frailty, sarcopenia and obesity within the CKD population, the transplant community is waking up to the need for prehabilitation and optimisation of patients on the waiting list. This audit provides granular data with respect to attitudes to exercise, which would be useful in designing prehabilitation programs and future studies in this area.

## Data Availability

The raw data supporting the conclusions of this article will be made available by the authors, without undue reservation.
